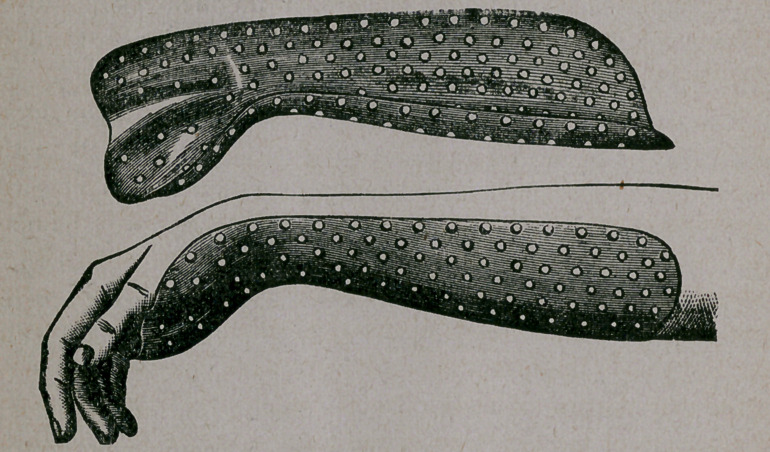# Levis’s Metallic Splints, for Fracture of Lower End of the Radius

**Published:** 1885-07

**Authors:** R. J. Levis

**Affiliations:** Surgeon to the Pennsylvania Hospital, and to the Jefferson College Hospital, Philadelphia, Pa.


### Selections

---

### Levis's Metallic Splints, for Fracture of Lower End of the Radius.

By R. J. Levis, M. D.

Surgeon to the Pennsylvania Hospital, and to the Jefferson College Hospital, Philadelphia, Pa.

The correct nature and mechanism of the ordinary form of fracture of the lower end of the radius is now, after much controversy, generally admitted and properly comprehended. With this proper understanding, the indications of treatment become rational and decisive.

In the usual and very characteristic fracture of the carpal end of the radius, the primary line of the fracture is, with little tendency to deviation, *transverse* in direction. Associated lines of fracture are generally those of comminution of the lower fragment, and are caused by the upper fragment being driven vertically into it and splitting it, usually in directions towards its articular surface.

The displacement of the lower fragment is towards the dorsal aspect of the forearm, and its articular surface is inclined in the same direction, abnormally presenting backwards and upwards.

The mechanism of the fracture is its production by falls upon the palm of the hand, which, with the carpus, undergoes extreme extension, and the fracture is caused by an *act of leverage* or *transverse strain*. This direction of force has also been called *cross-breaking strain*.

In this fracture, actual displacement of the lower fragment may not exist at all, or it may be to the extent of complete separation from contact of the broken surfaces, varying with the amount of force applied, and with the retaining influence of the surrounding dense structures.

The first essential of the treatment of fracture of the lower end of the radius is *the complete reduction of the displacement.* The action of replacement must be directed to the lower fragment itself. The reduction of the fracture can usually be thoroughly effected, under anesthesia, by *strong extension applied to the hand, associated with forced flexion of the wrist, and with pressure applied directly on the dorsal surface of the lower fragment.* Unless vertical splitting or comminution of the lower fragment exists, the maintaining of partial flexion of the wrist, with pressure of pad on the dorsal surface of the fragment, will prevent return of deformity.

With the object of retaining the apposition of the fractured surfaces, by overcoming displacing forces, I have practised for many years on the principles involved in the splint here illustrated, the application of which will not require much description.

In the treatment of fracture of the lower end of the radius, it is essential that proper allowance be made for the curvature of the anterior or palmer surface of this part of the bone. This is insured in the splint which I have devised, which follows correctly the radial curvature; and the fixing of the thenar and hypothenar eminences of the hand in their moulded beds, maintains the splint immovably in its erect position with reference to the radial curve.

To neglect of complete primary reduction of the displacement of the lower fragment, and to inefficient restoration and retention of the normal radial curve, are due the frequent unfortunate sequences of this fracture.

This splint is made of copper, so as to be readily comfortable by bending to suit the peculiarities of size and form of forearms. The slight roughness left on back of splint for perforations is for the purpose of keeping the bandage from slipping. It is nickel-plated to prevent oxidation.

The splint will usually fit the forearm so accurately that but little padding will be required, and a piece of woven lint, or of cotten or woolen flannel is all that is necessary for its lining. No dorsal splint is needed, but, as before referred to, a small pad will, in most cases, be required for the dorsal surface of the lower fragment. For retention of the splint, an ordinary bandage, two inches and a half to three inches wide, is all that is necessary.

This splint has the merits of being applicable to all cases of fracture of the lower end of the radius, and also to many other injuries involving the forearm and wrist, and, as now supplied, is very inexpensive.

These splints are made in two sizes, for adults and children, and are rights and lefts. Price, $1.00 per splint. They are manufactured by J. Ellwood Lee, Conshohocken, Pa.

## Figures and Tables

**Figure f1:**